# A Randomized Controlled Trial of Innovative Postpartum Care Model for Mother-Baby Dyads

**DOI:** 10.1371/journal.pone.0148520

**Published:** 2016-02-12

**Authors:** Corinne Laliberté, Sandra Dunn, Catherine Pound, Nadia Sourial, Abdool S. Yasseen, David Millar, Ruth Rennicks White, Mark Walker, Thierry Lacaze-Masmonteil

**Affiliations:** 1 Children’s Hospital of Eastern Ontario Research Institute, Ottawa, Ontario, Canada; 2 Better Outcomes Registry & Network Ontario, Ottawa, Ontario, Canada; 3 Department of Paediatrics, University of Ottawa, Ottawa, Ontario, Canada; 4 Department of Family Medicine, University of Ottawa, Ottawa, Ontario, Canada; 5 Obstetrics and Maternal Newborn Investigations, the Ottawa Hospital Research Institute, Ottawa, Ontario, Canada; 6 Department of Obstetrics and Gynecology, University of Ottawa, Ottawa, Ontario, Canada; University of Tennessee Health Science Center, UNITED STATES

## Abstract

**Objective:**

To evaluate the efficacy, safety, and maternal satisfaction of a newly established integrative postpartum community-based clinic providing comprehensive support for mothers during the first month after discharge from the hospital. Our primary interests were breastfeeding rates, readmission and patient satisfaction.

**Methods:**

A randomized controlled trial was conducted in Ottawa, Canada, where 472 mothers were randomized via a 1:2 ratio to either receive standard of care (n = 157) or to attend the postpartum breastfeeding clinic (n = 315). Outcome data were captured through questionnaires completed by the participants at 2, 4, 12 and 24 weeks postpartum. Unadjusted and adjusted logistic regression models were conducted to determine the effect of the intervention on exclusive breastfeeding at 12 weeks (primary outcome). Secondary outcomes included breastfeeding rate at 2, 4 and 24 weeks, breastfeeding self-efficacy scale, readmission rate, and satisfaction score.

**Results:**

More mothers in the intervention group (*n* = 195, 66.1%) were exclusively breastfeeding at 12 weeks compared to mothers in the control group (*n* = 81, 60.5%), however no statistically significant difference was observed (OR = 1.28; 95% CI:0.84–1.95)). The rate of emergency room visits at 2 weeks for the intervention group was 11.4% compared to the standard of care group (15.2%) (OR = 0.69; 95% CI: 0.39–1.23). The intervention group was significantly more satisfied with the overall care they received for breastfeeding compared to the control group (OR = 1.96; 95% CI: 3.50–6.88)).

**Conclusion:**

This new model of care did not significantly increase exclusive breastfeeding at 12 weeks. However, there were clinically meaningful improvements in the rate of postnatal problems and satisfaction that support this new service delivery model for postpartum care. A community-based multidisciplinary postpartum clinic is feasible to implement and can provide appropriate and highly satisfactory care to mother-baby dyads. This model of care may be more beneficial in a population that is not already predisposed to breastfeed.

**Trial Registration:**

ClinicalTrials.gov NCT02043119

## Introduction

Postnatal care is of the utmost importance for women and newborns as it assures a smooth transition from hospital to home and improves patient health outcomes [1–2]. Unfortunately, comprehensive postpartum support is not widely available for many mothers, potentially leading to unidentified health problems. Among them, breastfeeding difficulties can cause neonatal jaundice, dehydration and poor weight gain, resulting in emergency room visits, longer hospital stays, early hospital readmissions and breastfeeding cessation [3–4]. In jurisdictions such as New Zealand, Australia, Canada and the UK, home-visits done by nurses, midwives and peers are available for mother-baby dyads and have been studied thoroughly in the past decade [5–8]. Home-visits, while recognized for achieving high maternal satisfaction, also produce equivalent clinical outcomes for mothers and newborns as hospital-based follow-up [9–10]. Postpartum home-visits may also decrease newborn hospital admissions [11]. However, these visits are costly, and not always universally accessible [9]. In addition, they are not necessary in order to identify mothers needing additional postpartum support: other methods, such as telephone calls may be as effective [12–14].

Another service delivery model proposed for postpartum care is one where mothers and their newborn are offered to attend a community-based clinic to receive multidisciplinary care, since “multi-intervention and multidisciplinary approaches have the most profound effect on raising breastfeeding rates and improving health outcomes” [15]. A 2014 clinical expert’s article in *Obstetrics & Gynecology* suggested “integration of care among the obstetrician, pediatric provider, and lactation consultant may enable more women to achieve their breastfeeding goals, thereby improving health outcomes across two generations.” [16]. This statement epitomizes the underlying principles of the present study funded by the Ministry of Health of the Province of Ontario, Canada. In our study, we wished to evaluate the efficacy, safety, and maternal satisfaction of a newly established integrative postpartum community-based clinic providing comprehensive support for mothers during the first month after early discharge from the hospital. This multi-disciplinary clinic included family physicians, registered nurses and lactation consultants working together to assist mothers and offer postnatal care when needed. The objective of our study is to evaluate a comprehensive postpartum service in a community compared to hospital based care on breastfeeding rates, maternal satisfaction, and readmission.

## Patients and Methods

### Participants

A pilot study was first conducted, prior to the opening of the postpartum clinic, to assess feasibility and recruitment. It was determined that the number of deliveries at our catchment hospitals was approximately 6400/year, of which we estimated half would be eligible.

Participants from the current study were screened and recruited at the two campuses of The Ottawa Hospital between January and July 2014. Mothers were eligible if they were admitted to the birthing unit at either campus, had delivered a healthy singleton infant at a gestational age of >36+6 weeks, were ≥ 18 years old, with no diagnosed medical problems, were breastfeeding their baby and intended to continue upon discharge, and could be contacted by phone or email after hospital discharge. Women were excluded if they did not speak English or French, were unable to present to the clinic (transport not available), had birthed multiples or preterm babies, had no plan and desire to breastfeed, were adoptive mothers, had breast surgery or had been identified with a psychological risk that may impede their ability to attend the first appointment at the clinic. Out-of-province patients were also excluded given the geographic distance and difference in social services.

### Design and procedure

The study was approved by the Research Ethics Board at both The Ottawa Hospital and the Children’s Hospital of Eastern Ontario (CHEO). Eligible mothers were approached by a research assistant at any point during their stay in the mother-baby unit and received a thorough description of the study. After written informed consent was obtained and the patient was enrolled, baseline data was collected from the mother’s and the newborn’s hospital charts. Randomization was performed after confirmation by the attending health care provider that participants were healthy and ready for discharge. Study participants were randomly assigned to either the intervention (discharge with follow-up at the postpartum clinic) or the control (standard of care) groups via a 2:1 allocation ratio. Stratification was used to improve allocation balance by parity, type of delivery and hospital recruitment campus. Group designation was given from a randomization list, which was generated using a permuted randomized block design, with permutation block sizes of 3, 6, and 9 units, prior to study initiation by an external statistician (Dr. Franco Momoli). This list was entered directly into the data management system (REDCap)’s group allocation component all the while blinding the study researchers, recruiters, and participants to the randomization allocations prior to patient randomization and enrolment into the trial. The research assistants then informed the participant of their randomization group and gave appropriate information about the location of the postpartum clinic if assigned to it, as well as book the first clinic visit. Therefore, this was a non-blinded study with research staff, clinicians and study participants aware of their group allocation post randomization. Follow-up data was collected from all mothers at 2, 4, 12 and 24 weeks postpartum via a self-report web-based survey (REDCap) or a telephone interview conducted by a dedicated research assistant. This was felt to be a reliable method for data collection since studies have shown that maternal recall is valid and reliable for up to 3 years [17, 18]. As part of current standard of care practice, all women were contacted by phone at 3 weeks by a nurse from Ottawa Public Health to administer the Edinburgh Postnatal Depression Scale.

### Participant allocation groups (study arms)

Participants allocated to the control group were discharged according to current hospital standards and as per their physician’s or midwife’s decision. After hospital discharge, the participant and her baby were entitled to receive follow-up care and seek currently available breastfeeding support in the community (e.g. through their family doctor, Public Health Unit or private services), but could not attend the postpartum clinic.

Participants allocated to the intervention group were also discharged according to current hospital standards and were required to attend a pre-booked appointment at the postpartum clinic, scheduled within 48 hours of their discharge. Clinic staff followed up with participants if they failed to keep the mandatory follow-up appointment. This first appointment included maternal assessment and care (e.g., wound care, prescriptions), neonatal care (e.g., weight gain assessment, jaundice screening using transcutaneous bilirubinometer), blood work including total serum bilirubin (TSB), and breastfeeding assessment and support. Family physicians were available for on-site consultations in the mornings, and lactation consultants and registered nurses were at the clinic throughout the day from Monday to Friday and Saturday mornings. The Bilirubin Pathway, established by the Champlain Maternal Newborn Regional Program (CMNRP) [19], was used to guide the management of neonatal jaundice. Those guidelines provide recommendation for subsequent TSB and or trans-cutaneous bilirubin follow-up, according to the modified Buthani’s normogram and whether risk factors for hyperbilirubinemia are present or not [20]. For babies discharged before 24 hours, blood for the newborn screening was drawn at the first visit and sent by mail to the Newborn Screening Ontario laboratory located at our local children’s hospital. Additional follow-up visits were offered to participants as clinically indicated and as many times as they desired up to a maximum of six weeks following the birth of their baby.

### Outcome measures

#### Baseline data

Research assistants collected baseline data from the mother’s and newborn’s hospital charts once consent was obtained. This included recruitment campus, parity, delivery type (stratification criteria), as well as date and time of birth, gestational age, sex of baby, Apgar score, birth weight, infant weight before discharge, bilirubin value, prolonged stay in the nursery due to causes such as hyperbilirubinemia, newborn screening completion, supplementation given in hospital, as well as date and time of discharge.

#### Exclusive Breastfeeding

The primary outcome of this study was **exclusive breastfeeding at 12 weeks post-birth** because health benefits of breastfeeding have been shown after as little as 3 months of exclusive breastfeeding [21,22]. We defined exclusive breastfeeding as the feeding of the infant’s mother’s breast milk only (including expressed breast milk) for at least 2 weeks prior to the collected outcome. We also collected data for exclusive breastfeeding within the previous 24 hours, as per the WHO definition [23]. Additional breastfeeding information regarding partial breastfeeding, expressed breast milk and formula feeding was collected in our breastfeeding questionnaire for additional sensitivity analysis.

#### Breastfeeding Self-Efficacy Scale

The short version Breastfeeding Self- Efficacy scale (BSES) is a 14-item self-report instrument, which was used at 2, 4 and 12 weeks post-partum. Items are rated on a 5-point scale. The higher the score, the more confident a mother is in her ability to breastfeed. This is a reliable and validated tool [24] used to identify and recognize mothers who are less confident with various aspects of breastfeeding. It can also help identify mothers who are prone to discontinue breastfeeding early and who may require additional intervention and support to ensure success [24].

#### Postpartum Depression Scale

The Edinburgh Postpartum Depression Scale (EPDS) is a 10-item questionnaire used at 3 weeks postpartum which assesses mothers’ feelings during the week prior to test administration [25,26]. The EPDS is a valuable and efficient screening tool to identify patients at risk for “perinatal” depression. A score of 10 or greater indicates a possible depression. Mothers who score above 13 are likely to be suffering from a depressive illness of varying severity. In the event of scores >10, the Public Health Nurse administering the survey would refer the mother with a high score to appropriate services.

#### Socio-Demographics Survey

This survey was developed and adapted from questions and categories used in the Public Health Agency of Canada survey “What Mothers Say: The Canadian Maternity Experiences” [27]. It includes questions regarding the age range of the mother, education level, number of children, previously breastfed children as well as preferred method of transportation.

#### Postnatal problems and readmissions

We included questions in the participant survey that inquired on whether readmissions for mother or baby were necessary at each time-point, and if there were any visits to the Emergency Department.

#### Mother Satisfaction Survey

This 10 item survey was developed to evaluate the level of maternal satisfaction with the care received, with a focus on breastfeeding support received as well as communication with the healthcare professionals, access to the clinic and perception of its physical environment.

### Data analysis

#### Sample size

Based on an estimated 50% rate of exclusive breastfeeding at 12 weeks in the control group (Infant Care Survey 2005, City of Ottawa, Public Health Unit [28] and the pilot study we conducted prior to the RCT), using an equal sample comparison of proportions we estimate that 200 patients per group are needed to detect a 15% relative difference in the intervention group (α = 0.05, β = 0.9, one-sided test). Assuming an attrition rate of 15% (i.e. loss to follow up, unsuitability, or other unanticipated events), we set a target of 230 participants per group for a total of 460 women. Accounting for the logistical concern of making full use of the nursing staff, lactation consultants, and family physicians at the clinic, we decided to apply a 1:2 ratio for control to intervention allocations. Using an unequal sample comparison of proportions, we determined that 154 and 306 patients were needed for the control and intervention groups respectively to maintain the significance level, power and effect size outlined in the equal sample comparison.

#### Statistical Analysis

All outcomes were analyzed based on the intention-to-treat principle, in accordance with the randomization allocations described previously. The primary outcome of the study (exclusive breastfeeding at 12 weeks) was also analyzed using the as-treated population. The primary goal of this study was to test the hypothesis that rates of exclusive breastfeeding at 12 weeks (see outcome measures for definition) would be higher in the group of women attending the postpartum clinic, as compared to those under standard of care. We employed logistic regression to examine the effect of the intervention program on the week 12 exclusive breastfeeding rate. An unadjusted model (primary model) including the control vs. intervention group as well as an adjusted model (sensitivity analysis) adjusting for potential confounders such as mode of delivery, parity, recruitment site, and supplementation in the nursery, etc., on the primary outcome was conducted. Unadjusted and adjusted odds ratios and their respective 95% confidence intervals were produced.

Secondary outcomes were collected by reviewing the medical chart or through participant survey responses (breastfeeding at 2, 4 and 24 weeks, BSES, EPDS, satisfaction). Differences between the intervention and control group on these secondary outcomes were tested using univariate tests, Pearson Chi-squared or Student’s t depending on the nature of the outcome. Univariate tests were also used to compare baseline characteristics between the two groups. As these tests were not powered or pre-specified, no adjustment for multiple testing was performed on the p-values. Therefore any statistically significant findings for baseline comparisons or secondary outcomes should be considered hypothesis-generating and be interpreted with some caution. All analyses were conducted excluding missing values and using the SAS 9.4 software (Cary, NC).

## Results

### Participant characteristics and baseline

**Fig 1:**
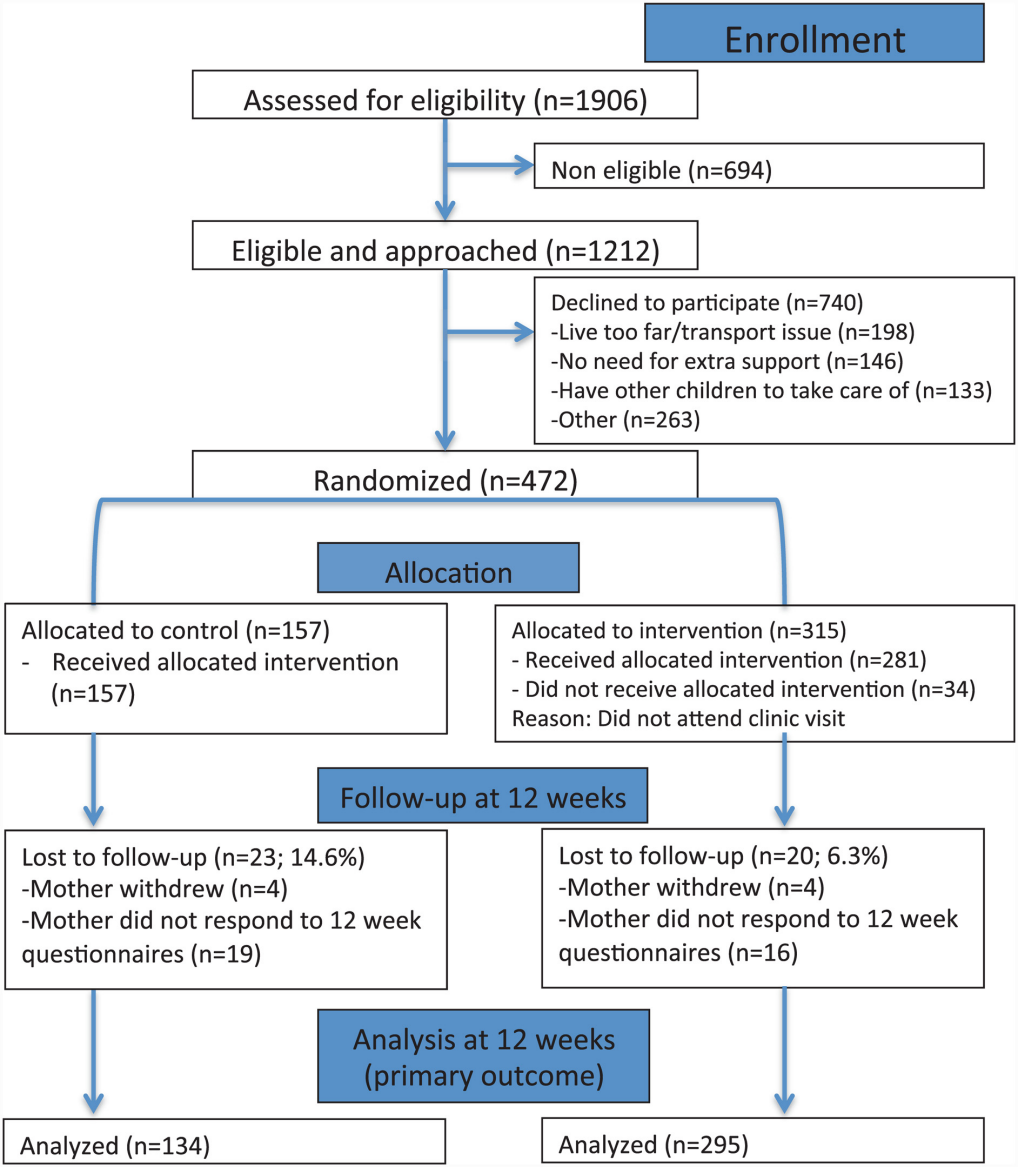
Flowchart of eligible and consented mothers.

**Fig 2:**
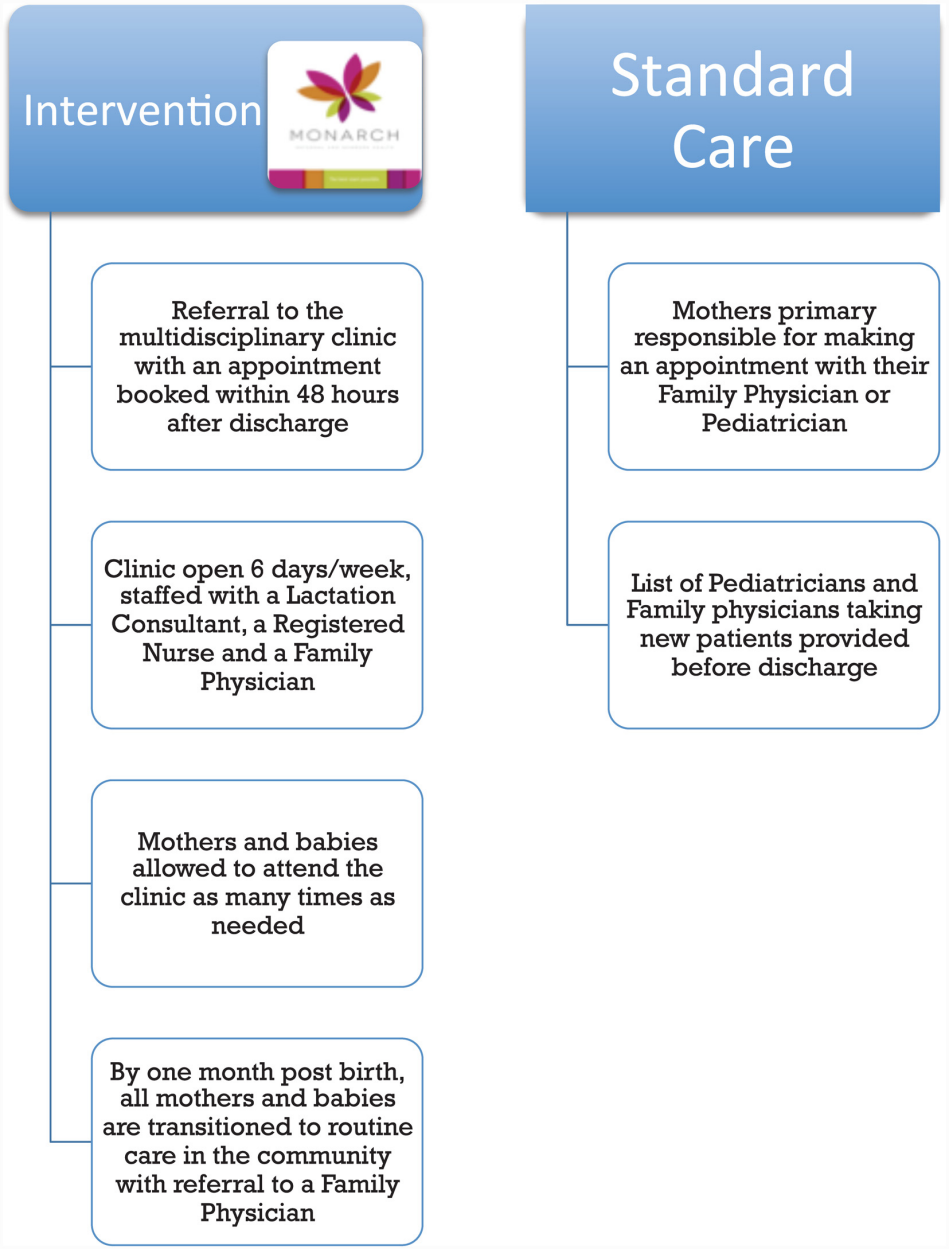
Flowchart comparing care received by the intervention group versus the standard of care group.

Of the 1,906 mothers screened, 694 (36.4%) were ineligible (Fig 1). Of the 1,212 potentially eligible mothers, 740 (61.1%) declined consent. The most common reasons for declining to participate are listed in Fig 1. A total of 472 consented to participate in the study; 315 mothers were randomized to the intervention and 157 mothers to the control group. Fig 2 demonstrates the differences in the intervention and standard of care groups. The majority of enrolled mothers were primiparous, married/common law, had completed university education, and were 30 years or older (Table 1).

**Table 1:** Mother Demographics Characteristics.

Mother Demographics	Intervention (*n* = 294), *n* (%)	Control (*n* = 134), *n* (%)
**Age range**		
15–19, y	1 (0.3)	1 (0.8)
20–24, y	16 (5.4)	6 (4.5)
25–29, y	67 (22.8)	28 (20.9)
30–34, y	105 (35.7)	60 (44.8)
35–39, y	76 (25.9)	32 (23.9)
40 +, y	21 (7.1)	5 (3.7)
*Missing*	8 (2.7)	2 (1.5)
**Married/common law**	272 (92.5)	125 (93.3)
*Missing*	7(2.4)	2 (1.5)
**Highest education level**		
Elementary school / Some high school	0 (0)	1 (0.8)
Completed high school	26 (8.8)	7 (5.2)
Vocational / technical training (post high school)	49 (16.7)	18 (13.4)
Completed university	212 (72.1)	104 (77.6)
*Missing*	7 (2.4)	4 (3.0)

One significant difference was present at baseline between randomized groups (Table 2): the control group had a 10.6% higher rate of supplementation given during hospital stay, compared to the intervention group (46.5% versus 35.9%, OR:0.64; 95% CI: 0.44–0.95), p = 0.03). There were no other significant differences noted between both groups (Table 2).

**Table 2:** Baseline Characteristics.

Baseline	Intervention (*n* = 315), *n* (%)	Control (*n* = 157), *n* (%)
**Maternal**		
Primiparous	195 (61.9)	97 (61.8)
C-Section	90 (28.6)	43 (27.4)
**Neonatal**		
Male sex	164 (52.1)	89 (56.7)
Gestational age, week	39.7 (1.2)	39.6 (1.3)
Birth weight, g	3505.6 (473.9)	3423.2 (444.0)
Weight before discharge, g	3289.7 (455.1)^a^	3219.3 (419.1)^b^
Apgar score < 8 at 5 min	10 (3.2)	4 (2.6)
Highest bilirubin,	136.4 (62.9)^c^	132.4 (60.7)^d^
Phototherapy required before discharge	29 (9.2)	10 (6.4)
Supplementation given during hospital stay	113 (35.9)	73 (46.5)
LOS^e^, after vaginal delivery, h	47.2 (23.4)	49.1 (25.0)
LOS^e^, after C-section, h	71.5 (20.0)	70.1 (13.5)

^a^ Missing values = 2, n = 313

^b^ Missing values = 1, n = 156

^c^ Missing values = 2, n = 313

^d^ Missing values = 2, n = 155

^e^ LOS: length of stay in hospital before discharge home

Follow-up data at 12 weeks was completed by a larger proportion of participants of the intervention group (93.7%, n = 295), compared to the control group (85.4%, n = 134) (Fig 1), with the majority of the lost-to follow-ups occurring at the first time-point (2 weeks postpartum).

### Visits at the postpartum clinic

Of the 315 mothers who were randomized at baseline to the intervention group, 34 (10.8%) did not attend the clinic. From the intervention group, 4 withdrew from the study and 16 were lost to follow-up at 12 weeks (primary outcome) (Fig 1). More than 50% of the mothers assigned to the intervention attended the clinic on at least 3 occasions (Table 3).

**Table 3:** Clinic Visit Information.

Total Clinic Visits	*n* (%)
0	34 (10.8)
1	56 (17.8)
2	62 (19.7)
3	64 (20.3)
4+	99 (31.4)

### Primary outcome

There was no significant difference in the rate of exclusive breastfeeding between the two groups at 12 weeks. The exclusive breastfeeding rate in the intervention group was 66.1% and that of the control group was 60.5% (OR: 1.28; 95% CI: 0.84–1.95). The difference remained non significant after controlling for mode of delivery, parity, recruitment site, and supplementation in the nursery (aOR (adjusted OR): 1.12; 95% CI: 0.72–1.75). Likewise, after performing an as-treated instead of intent-to-treat analysis, the difference remained in favour of the intervention group though non significant (OR: 1.20; 95% CI: 0.78–1.84).

Whereas there was no significant difference in the rate of exclusive breastfeeding at 12 weeks between the two groups in the multivariate model, C-section (versus vaginal) delivery (aOR: 1.65; 95% CI: 1.02–2.69, p = 0.04), multiparous (versus primiparous) (aOR: 1.59 95% CI: 1.02–2.47, p = 0.04), and no formula supplementation in the nursery (versus any supplementation) (aOR: 3.44 95% CI: 2.23–5.32, p <0.01) were significantly associated with exclusive breastfeeding at 12 weeks.

For mothers exclusively feeding directly from the breast, there was a 7.1% difference, although non-statistically significant, in favor of the intervention group (33.2% vs 26.1%), OR: 1.41; 95% CI: 0.89–2.22) We also compared exclusive breastfeeding rates as defined by the WHO (breastfeeding in the previous 24 hours) which showed similar trends [20]; therefore this data is not presented. Table 4 indicates breastfeeding rates at 2, 4, 12 and 24 weeks for both groups as well as the proportion of women who ceased breastfeeding completely. Similar trends in favor of the intervention group were observed at all time points, but none of the differences were found to be statistically significant.

**Table 4:** Breastfeeding outcomes at 2, 4, 12 and 24 weeks postpartum.

Outcome	Intervention, *n* (%)	Control, *n* (%)	*Odds Ratio*
**Week 2**			
Infants exclusively breastfeeding in previous 2 weeks	192/295 (65.1)	82/140 (58.6)	1.32 (0.87–1.99)
Infants partially breastfed in previous 2 weeks	86/295 (29.2)	45/140 (32.1)	0.87 (0.56–1.34)
Infants exclusively formula-fed in previous 2 weeks	15/295 (5.1)	10/140 (7.1)	0.70 (0.30–1.59)
Missing	2/295 (0.7)	3/140 (2.1)	N/A
Total	295/295 (100)	140/140 (100)	N/A
Infants exclusively breastfeeding ***at the breast*** in previous 2 weeks	97/295 (32.9)	35/140 (25.0)	1.47 (0.93–2.31)
Infants breastfeeding any amount in previous 2 weeks	266/295 (90.2)	119/138 (86.2)	1.46 (0.79–2.72)
**Week 4**			
Infants exclusively breastfeeding in previous week	191/294 (65.0)	80/134 (59.7)	1.25 (0.82–1.91)
Infants partially breastfed in previous week	85/294 (28.9)	43/134 (32.1)	0.86 (0.55–1.34)
Infants exclusively formula-fed in previous week	16/294 (5.4)	9/134 (6.7)	0.80 (0.34–1.86)
Missing	2/294 (0.7)	2/134 (1.5)	N/A
Total	294/294 (100)	134/134 (100)	N/A
Infants exclusively breastfeeding ***at the breast*** in previous week	96/294 (32.7)	33/134 (24.6)	1.48 (0.93–2.36)
Infants breastfeeding any amount in previous week	264/294 (89.8)	115/133 (86.5)	1.38 (0.74–2.57)
**Week 12 (Primary outcome)**			
**Infants exclusively breastfeeding in previous 2 weeks**,	**195/295 (66.1)**	**81/134 (60.5)**	**1.28 (0.84–1.95)**
Infants partially breastfed in previous 2 weeks	84/295 (28.5)	43/134 (32.1)	0.84 (0.54–1.31)
Infants exclusively formula-fed in previous 2 weeks	14/295 (4.8)	8/134 (6.0)	0.78 (0.32–1.92)
Missing	2/295 (0.7)	2/134 (1.5)	N/A
Total	295/295 (100)	134/134 (100)	N/A
Infants exclusively breastfeeding ***at the breast*** in previous 2 weeks	98/295 (33.2)	35/134 (26.1)	1.41 (0.89–2.22)
Infants breastfeeding any amount in previous 2 weeks	267/295 (90.5)	117/132 (88.6)	1.22 (0.63–2.37)
Mothers ceased breastfeeding	30/307 (9.8)	19/145 (13.1)	0.69 (0.37–1.28)
Missing	10/307 (3.3)	9/145 (6.2)	N/A
**Week 24**			
Infants exclusively breastfeeding in past 2 weeks	151/292 (51.7)	64/138 (46.4)	1.24 (0.83–1.86)
Infants received breast milk at the breast in past 2 weeks	231/292 (79.1)	109/138 (79.0)	1.01 (0.61–1.66)
Infants some received breast milk or EBM^a^ in past 2 weeks	242/292 (82.9)	112/138 (81.2)	1.12 (0.67–1.90)
Introduced solid food	199/291 (68.4)	91/138 (65.9)	1.12 (0.73–1.72)

^a^ EBM = Expressed Breast Milk

### Secondary outcomes

Tables 5, 6 and 7 show the secondary outcomes. No significant differences were seen between groups for the mean Edinburgh Postpartum Depression Score at 3 weeks. There were no significant differences between groups for the mean breastfeeding self-efficacy scores at 2, 4 and 12 weeks. Self-reported rates of ER visits and readmissions (Table 6) were not significantly lower in the intervention group compared to the control group. The rate of ER visits at 2 weeks for the intervention group was 11.4% compared to the standard of care group (15.2%) (OR = 0.69; 95% CI: 0.39–1.23). The intervention group was significantly more satisfied with the overall care they received for breastfeeding compared to the control group (mean score: 45.0 versus 50.2; OR: 1.96; 95% CI: 3.50–6.88) (Table 7).

**Table 5:** Secondary outcomes on Breastfeeding Self-Efficacy Scale and Edinburgh Postpartum Depression Score.

Variable	Intervention, mean (SD)	Missing, n(%)	Control, mean (SD)	Missing, n(%)
BSES^a^ at 2 weeks	55.7(12.2) n = 295	36(12.2)	55.1(11.4) n = 140	25(17.9)
BSES at 4 weeks	55.8(12.3) n = 294	35(11.9)	55.5(11.4) n = 134	21(15.7)
BSES at 12 weeks	55.9(12.3) n = 295	33(11.2)	55.4(11.2) n = 134	20(14.9)
EPDS^b^ response rate, n (%)	217 (68.9%)	98(31.1)	61 (38.9%)	96(61.1)
EPDS score at 3 weeks	4.47(3.49)	98(31.1)	4.67(2.65)	96(61.1)

^a^ Breastfeeding Self-Efficacy Scale

^b^ Edinburgh Postpartum Depression Score

**Table 6:** Secondary outcome for use of healthcare resources.

Variable	Intervention, n(%)	*Missing*, *n(%)*	Control (n =), n(%)	*Missing*, *n(%)*	Odds ratio
Self-reported ER^a^ visit at 2 weeks^b^	35/307(11.4)	16/307(5.2)	22/145(15.2)	12/145(8.3)	0.69(0.39–1.23)
Self-reported ER visit at 4 weeks^c^	17/307(5.5)	17/307(5.5)	11/145(7.6)	16/145(11.0)	0.67(0.30–1.47)
Self-reported ER visit at 12 weeks^d^	31/307(10.1)	15/307(4.9)	14/145(9.7)	18/145(12.4)	0.96(0.49–1.87)
Total participants with at least one reported ER visit	63/307(20.5)	33/307(10.8)	26/145(17.9)	30/145(20.7)	1.02(0.61–1.72)
Self-reported readmission of infants at 2 weeks^b^	18/307(5.9)	17/307(5.5)	8/145(5.5)	11/145(7.6)	1.04(0.44–2.46)
Self-reported readmission of infants at 4 weeks^c^	6/307(2.0)	21/307(6.8)	4/145(2.8)	16/145(11.0)	0.67(0.19–2.41)
Self-reported readmission of infants at 12 weeks^d^	8/307 (2.6)	15/307(4.9)	4/145(2.8)	17/145(11.7)	0.87(0.26–2.95)
Total participants with at least one reported infant readmission	20/307(6.5)	37/307(12.1)	9/145(6.2)	27/145(18.6)	0.97(0.43–2.20)
Self-reported readmission of mothers at 2 weeks^b^	10/307(3.3)	15/307(4.9)	5/145(3.5)	8/145(5.5)	0.94(0.31–2.79)
Self-reported readmission of mothers at 4 weeks^c^	4/307(1.3)	17/307(5.5)	2/145(1.4)	16/145(11.0)	0.89(0.16–4.91)
Self-reported readmission of mothers at 12 weeks^d^	8/307(2.6)	15/307(4.9)	1/145(0.7)	17/145(11.7)	3.58(0.44–28.90)
Total participants with at least one reported mother readmission	13/307(4.2)	33/307(10.8)	5/145(3.5)	25/145(17.2)	1.15(0.40–3.29)

^a^ Emergency Room Visit

^b^ This is the time-point at which the questionnaire was administered. The period covered is from birth to week 2.

^c^ This is the time-point at which the questionnaire was administered. The period covered is from week 2 to week 4.

^d^ This is the time-point at which the questionnaire was administered. The period covered is from week 4 to week 12.

**Table 7:** Maternal Satisfaction.

Satisfaction criteria	Intervention (*n* = 295), % very satisfied and satisfied, mean (SD)	Missing values, n(%)	Control (*n* = 134), % very satisfied and satisfied, mean (SD)	Missing values, n(%)	*Odds Ratio*
Satisfied with amount of information given by HCP^a^	88.5 (68.8+19.7)	15(5.1)	80.6 (42.5+38.1)	4(3.0)	2.80 (1.46–5.38)
Satisfied with opportunities to ask questions	88.5 (75.3+13.2)	12(4.1)	62.7 (37.3+25.4)	4(3.0)	6.50 (3.69–11.42)
Satisfied with opportunities to give opinion	74.5 (60.3+14.2)	11(3.7)	65.6 (44.0+21.6)	4(3.0)	1.64 (1.03–2.60)
Satisfied with availability shown by HCP^a^	88.2 (70.9+17.3)	13(4.4)	76.2 (47.8+28.4)	4(3.0)	3.24 (1.77–5.93)
Satisfied with breastfeeding support received	87.5 (68.5+19.0)	13(4.4)	64.2 (32.1+32.1)	4(3.0)	5.50 (3.16–9.57)
Satisfied with support received while transitioning from hospital to home	84.4 (62.7+21.7)	12(4.1)	72.4 (38.8+33.6)	3(2.2)	2.57(1.51–4.36)
Total general satisfaction score	50.2 (6.9)	25(8.5)	45.0(8.4)	10(7.5)	P <0.0001
If a breastfeeding clinic that offered maternal care, neonatal care and breastfeeding support were to be available, do you think that would be useful for new mothers?	93.3 (84.8 + 8.5)	13(4.4)	91.8 (80.6 + 11.2)	3(2.2)	2.56 (0.91–7.20)
**Clinic participants only**					
Satisfied with the access to the breastfeeding clinic (location and transportation)	83.4 (59.3+24.1)	11(3.7)	N/A	N/A	N/A
Satisfied with the physical environment of the breastfeeding clinic (noise, comfort, privacy)	88.8 (72.9+15.9)	13(4.4)	N/A	N/A	N/A
Satisfied with the opening hours of the clinic	84.4 (70.8+13.6)	12(4.1)	N/A	N/A	N/A
Satisfied with the easiness with which you could get an appointment at a convenient time for you at the clinic	84.7 (72.2+12.5)	13(4.4)	N/A	N/A	N/A

^a^ HCP: Healthcare professional

### Association between BSES scores and the primary outcome

Although there were no significant differences between groups for the mean breastfeeding self-efficacy scores at 2, 4 and 12 weeks, there were statistically significant differences between the mean BSES score at 2 weeks for women in both the intervention and control groups and exclusivity of breastfeeding at 12 and 24 weeks postpartum. Women in both groups with higher BSES scores at 2 weeks were significantly more likely to be exclusively breastfeeding at 12 and 24 weeks postpartum than women with lower BSES scores (Table 8).

**Table 8:** Association between BSES scores and the primary outcome.

	Mean BSES Score at 2 weeks (SD)		
**Variable**	Exclusive breastfeeding in past 2 weeks	Not exclusive breastfeeding in previous 2 weeks	P-value
**12 weeks**			
Intervention	52.3 (10.7)	36.8 (12.8)	0.0001
Control	50.8 (11.8)	37.9 (14.5)	0.0001
Total	51.9 (11.1)	37.2 (13.4)	0.0001
**24 weeks**			
Intervention	50.3 (11.5)	43.5 (14.9)	0.0001
Control	48.8 (11.7)	44.1 (15.3)	0.0520
Total	49.8 (11.6)	43.7 (15.0)	0.0001

## Discussion

In this randomized controlled trial comparing mothers receiving standard of care and mothers attending a postpartum community-based clinic post-discharge, there was no significant difference in exclusive breastfeeding rates at 12 weeks postpartum. The exclusive breastfeeding trend was stable over time in the intervention group, and was recorded at 65.1%, 65.0% and 66.1% at week 2, week 4 and week 12 respectively. The exclusive breastfeeding rates in the control group were similarly stable: 58.6%, 59.7% and 60.5% at week 2, week 4 and week 12 respectively. Exclusive breastfeeding rates were found to be higher at 24 weeks (51.7%) in the intervention group than in the control group (46.4%) but this did not achieve statistical significance. A significant difference was found in maternal satisfaction, with the intervention group showing a mean satisfaction score of 50.2 (6.9), as compared to a mean score of 45.0 (8.4) in the control group.

Our inability to show a significant difference in primary outcome could be related to the demographic characteristics of our population. The majority of enrolled mothers were more than 29 years old and very educated, with almost ¾ having attended University (in 2009, around 37.2% and 28.1% of all Canadian women aged 25 to 54 had a post-secondary certificate/diploma and a University degree respectively [29]). This is consistent with other studies of breastfeeding women in our area [30], and may be due to the fact that women who are older and more educated tend to breastfeed longer [31–33]. This is evidenced by the proportion of women in our control group who were breastfeeding at 3 months (60.5%), higher than that of the general Ontario population (52.6%) [34]. Furthermore, breastfeeding self-efficacy scores were found to be stable though the study period, with mean BSES scores approaching 56 for both groups. This suggests our participants had “high efficacy” [35], and supports our hypotheses that both groups consisted of highly motivated mothers. This may have attenuated the expected difference in breastfeeding rates.

Interestingly, baseline data revealed a significantly higher in-hospital supplementation rate in the control group (46.5%) as compared to the intervention group (35.9%). This difference in the randomized groups makes the initial populations heterogeneous. This could be due to chance but could also be due to the non-blinded study design, for clinicians and the patients’ circle of care. Physicians and nurses of patients randomized to the clinic may have felt less concerned with the infant’s feeding pattern, knowing that there would be follow-up with access to lactation consultants shortly after discharge, and therefore may have decreased their likelihood of introducing formula supplementation. The overall predischarge supplementation rate for the Province of Ontario in year 2014–2015 is 35.2% (BORN Ontario, Better Outcomes Registry and Network). As suggested by others [36], the logistic regression analysis of our study confirms that early supplementation is associated with lower rate of exclusive breastfeeding at 12 weeks post birth. Such a high percentage of supplementation has been identified by the Government of Ontario as a serious health issue and is currently the focus of the Ministry of Health Baby Friendly Initiative strategy.

We can also draw a parallel between the high motivation and the breastfeeding self-efficacy scores, which were stable throughout the study period. An interesting and significant finding was related to BSES scores and exclusivity of breastfeeding. When we compared BSES scores at 2 weeks postpartum with breastfeeding exclusivity at 12 and 24 weeks postpartum, mean scores for mothers who had exclusively breastfed (or fed expressed milk) in the previous 2 weeks were significantly higher than the mean scores for the mothers who had not exclusively fed their infants breastmilk. This trend held true for both the intervention and control groups. BSES scores in the early postpartum period are predictive of breastfeeding duration at 4, 6, 8 and 16 weeks postpartum with lower scores being associated with early discontinuation of breastfeeding [37, 38]. Therefore, our results are also clinically significant and suggest that measuring BSES early in the postpartum period could provide important information for clinicians to help them identify mothers requiring additional supports (those with lower BSES), and to provide information to design of tailored supports for women who have initiated breastfeeding [38].

A large proportion of mothers in the intervention group (71.4% (n = 225)) attended the clinic more than once, even though only one visit was mandatory for the study. This suggests that mothers were satisfied with the care provide at the clinic and returned for additional services. This hypothesis is supported by the results of our maternal satisfaction questionnaire administered at 12 weeks. The intervention group’s level of satisfaction was much greater (significant for all criteria shown in Table 7) than that of the control group. The most significant items on the questionnaire were the following: “satisfaction with the opportunity to ask questions”, “satisfaction with breastfeeding support received” and “satisfaction with support received while transitioning from hospital to home”, potentially identifying areas where the current standard of care is lacking. These results are similar to those obtained in another breastfeeding study conducted in the Ottawa area, showing an increased in maternal satisfaction when more support is provided [39]. With higher levels of support, mothers described a “higher level of empowerment and emotional wellbeing” [39], with positive effects on breastfeeding experience and potentially duration. Areas of dissatisfaction for women enrolled in the intervention group included “access to the breastfeeding clinic (location and transportation)”, with 6.1% of clinic attendees responding negatively (n = 18), suggesting that having more locations for postpartum clinics could be a solution to accessibility for mothers and their newborns.

Limitations to this study are threefold. First, the study may have been underpowered. We achieved our study power for the desired significance and effect size, however, there is a possibility that our effect size (i.e. a 15% difference) might be too large. The power calculation was based on detecting a 15% difference assuming an estimated rate of 50% in the control group. Because the actual rate in the control group was 10% higher than expected, the actual effect size was only 6%. A larger study sample might have been able to detect a significant difference. A second limitation is that our findings may not be generalizable to countries with shorter lengths of stay following birth, such as the UK. A third limitation is that it is possible that through the selection process, we may have excluded a fraction of the population (i.e. mothers not able to transport to the clinic, living too far, etc.) that could have benefitted from the intervention. A fourth limitation was the response rate for the Edinburgh Postpartum Depression Score. A much larger proportion of the intervention group (68.9%) responded to the EPDS compared to the control group (38.9%), therefore no conclusions were made regarding this aspect of the study. The final limitation is related to the lack of a cost-effectiveness component. Our surveys did gather some information on the use of resources in the community, however a more thorough analysis of cost is warranted. In the future, we will address this issue by linking the study dataset to a set of Provincial administrative datasets to better understand the resource consumption and long-term effects of the intervention on the population. Finally, the Ottawa population of new mothers may not be the ones who would benefit the most from this type of clinic since the baseline breastfeeding rate is already quite high. It would be interesting to test this new model of care delivery in a more at-risk socioeconomic environment, where mothers attending the postpartum clinic could have greater gains than the socioeconomically-advantaged mothers in our study.

## Conclusion

The multidisciplinary postpartum clinic did not increase exclusive breastfeeding by a statistically significant amount at 12 weeks. It may be that our population was already very motivated to breastfeed from the onset, and that this particular delivery of care would show a greater effect in a more at-risk socioeconomic environment. Regardless, attendance at the clinic resulted in clinically meaningful gains, including an increase in maternal satisfaction level. Specific satisfaction items were identified as problematic in the current standard of care, and may suggest areas to target in the future with the development of strategies for postpartum care.

Additional research should be done with a more diverse population of mothers (education-level wise), different locations for this type of clinic and possibly additional services. Whether such a multidisciplinary postpartum clinic may allow mothers and babies to be early (less than 24 hours after vaginal delivery) discharged remains to be assessed.

## Supporting Information

S1 Checklist CONSORT Checklist. (DOC)

S1 Protocol Study Protocol. (PDF)
