# Supplementary material for: A Randomized Controlled Trial of Innovative Postpartum Care Model for Mother-Baby Dyads
Source: PLoS One. 2016 Feb 12;11(2):e0148520. doi: 10.1371/journal.pone.0148520 (PMC4752489; doi:10.1371/journal.pone.0148520)
Supplement: S1 Protocol — (PDF) [file pone.0148520.s002.pdf]

RESEARCH PROTOCOL

A Randomized Controlled Trial:

BEST ABCs: Benefits and Effectiveness of Support offered Through A Breastfeeding Clinic Study

Date of Version (#4): 10 June 2014

Principal Investigator: Dr. Thierry Lacaze-Masmonteil

BEST ABCs: Benefits and Effectiveness of Support offered Through A Breastfeeding Clinic Study: A randomized controlled trial

Date of version: 10 June 2014

PROTOCOL SIGNATURE PAGE

My signature below confirms that I have reviewed and approved this protocol, and agree that it contains all necessary details for carrying out the study as described. I will conduct this protocol as outlined therein, and according to Good Clinical Practice and all applicable local regulations.

Principal Investigator Name (Please Print)

Principal Investigator Signature

Date

Content

1 Background ... 5
2 Aim of Study and Study Design ... 6
2.1 Study Objectives ... 6
2.2 Hypothesis ... 7
2.3 Primary Outcome ... 7
2.4 Secondary Outcomes ... 7
2.5 Inclusion criteria: ... 7
2.6 Exclusion criteria: ... 8
3 Methods: ... 8
3.1 Participant recruitment and informed consent ... 8
3.2 Events prior to hospital discharge ... 8
3.2.1 Data collection ... 8
3.2.2 Randomization ... 8
3.2.3 Appointment booking and additional information given to the participant (intervention arm) 9
3.3 Non-intervention arm post-discharge events ... 10
3.4 Intervention arm post-discharge events ... 10
3.5 Follow-up surveys at 2 weeks, 1 month and 3 months after hospital discharge ... 11
3.6 Risks and risk management ... 13
3.7 Criteria for removal/withdrawal from the study ... 13
3.8 Study calendar ... 13
4 Statistics ... 14
4.1 Sample size ... 14
4.2 Feasibility ... 14
4.3 Statistical analysis ... 15
5 Data management ... 16
5.1 Data collection ... 16
5.2 Database security ... 16
6 Ethical considerations ... 17
6.1 Ethics committee ... 17
6.2 Participant information and consent ... 18
6.3 Compensation ... 18

6.4 Records retention 18
7 Reference List 18
8 Evaluation tools 19
8.1 Demographics 19
8.2 Edinburgh Post Partum Depression Scale 19
8.3 Breastfeeding Self Efficacy Scale 19
8.4 Local Breastfeeding Complications Questionnaire 19
8.5 Use of Resources Questionnaire 19
8.6 Breastfeeding Questionnaire 19
8.7 Mother Satisfaction Survey 19
8.8 Informative pamphlet 19
8.9 Recruitment flyer 19

1 Background

While breastfeeding initiation rates are high in Ontario, duration and exclusivity rates drop precipitously in the first weeks and months after birth. Despite being above the national average, Ottawa breastfeeding rates are still low, with 56.3% of women exclusively breastfeeding shortly after birth, down to 39.1% at 6 months according to an Infant Care Survey.[1] In Canada, the exclusive breastfeeding rate at 6 months, the widely accepted ideal standard to achieve, was reported to be 14.1% in the Canadian Maternity Experience Survey.[2] Breastfeeding confers a multitude of long-term infant and maternal health benefits, with the potential for major economic gains. Studies have shown that each additional month of exclusive breastfeeding can lead to a 30% decrease in infection-related hospitalizations in the first few months of life.[3] Also, formula-fed healthy infants experience a three times higher risk for developing severe respiratory illness when compared to infants exclusively breastfed for 4 months.[4] Moreover, breastfeeding duration during infancy was found to be inversely proportional to the risk of being overweight in childhood.[5] For mothers, breastfeeding decreases the risk of breast [6,7] and ovarian cancers.[8] Also, an inverse relationship between maternal depressive symptoms and breastfeeding has been shown.[9] Therefore, health dollars invested in supporting and promoting exclusive breastfeeding have the potential to yield a high rate of return in preventing acute and chronic illnesses. Low breastfeeding self-efficacy [10] , insufficient knowledge, skills and support, and breastfeeding problems that lead to inadequate milk supply are associated with early weaning . If these modifiable factors can be addressed early and consistently through support from lactation experts, the likelihood increases that a woman will breastfeed longer and more exclusively.[11] By providing mothers with breastfeeding support, tools and techniques, their confidence in their ability to breastfeed will increase, with direct positive repercussions on exclusive breastfeeding duration.

Breastfeeding support strategies that rely on face-to-face interventions are more likely to succeed than support offered reactively, as per a recent systematic review.[11] Women should be offered ongoing opportunities to receive advice through scheduled visits so as to ensure support availability. Unfortunately, comprehensive breastfeeding support is not widely available for many women, leading to unidentified breastfeeding difficulties. These difficulties can result in longer hospital stays and early hospital readmissions, because of their association with conditions such as neonatal jaundice, dehydration and poor weight gain. These neonatal concerns could, however, be addressed comprehensively in a community setting (and not in the hospital), resulting in a decrease in prolonged hospital stay and hospital readmissions. However, little is known about the most effective way to support and manage early postpartum issues, particularly jaundice, in the community.

We have received funding from the Ontario Ministry of Health and Long-Term Care to evaluate the efficacy and cost effectiveness of a breastfeeding/jaundice clinic based in the community after early discharge from the hospital. This multidisciplinary community clinic, developed in partnership between the research team, the Ottawa Hospital, and Public Health, will provide a comprehensive support for breastfeeding mothers during the first month after delivery. Open six days a week, the clinic will be staffed by family physicians (FP), Registered Nurses (RN), both health care providers with maternal/newborn experience, and Public Health Nurses (PHN)/lactation consultants (LC), to provide an initial assessment within 12 to 48 hours after hospital discharge and then follow-up breastfeeding consultations as needed during the first month after discharge. FP will be available each morning to do

an assessment of the infants and the mothers who are attending the clinic. The clinic will only enroll eligible women willing to participate in a research project to evaluate this new program, in order to assess whether early discharge with the support of this type of program in the community really helps breastfeeding mothers and their babies in transitioning from the hospital to home. The funding will support: 1) 1.0 FTE PHN/LC and 1.0 FTE RN on site, 2) the research infrastructure for a randomized controlled trial comparing the percentage of exclusive breastfeeding at 3 months and other secondary outcomes, between 2 groups of participants recruited at the Ottawa Hospital (e.g., women receiving the current standard of care and women accessing the services of the post-partum clinic).

The objective of the BEST ABCs trial will be to explore a new model for post-partum transition by offering women and their infants postpartum support in a multidisciplinary clinic. This clinic will address three important themes described in the literature: improving duration and rate of exclusive breastfeeding, preventing costly postnatal (maternal and neonatal) problems (e.g. the interrelated issues of jaundice and breastfeeding difficulties), and determining benefits associated with shorter hospital stays. For families that have challenges accessing services, even small problems can quickly manifest into difficulties leading to breastfeeding cessation, emergency room visits, and costly readmissions. The proposed community clinic will address early postpartum maternal newborn problems, move care away from hospitals when feasible and safe, and enhance health care system value. We will evaluate patient outcomes, mother satisfaction, and the costs associated with this new model. If successful, this model could be tested throughout Ontario.

We are currently conducting a pilot study (Phase 1) at The Ottawa Hospital over 3 months before the beginning of the BEST ABCs trial (Phase 2) to assess the feasibility of recruiting women and to test the data collection tools that we have developed and will be using for the RCT. We are collecting information on the current experience of breastfeeding women in the Ottawa region and their use of existing resources to provide a baseline estimate of the outcome rates.

2 Aim of Study and Study Design

2.1 Study Objectives

We will enroll women meeting the inclusion and exclusion criteria for the trial. The objectives of this study are to:

1. Determine if care provided through this community based postpartum clinic can improve duration and rate of exclusive breastfeeding, prevent costly postnatal problems and reduce length of hospital stay and the number of readmissions for new mothers.

2. Determine the level of satisfaction of the mothers attending this clinic with the care they received.

3. Determine the cost effectiveness of the new clinic and what the costs are associated with implementing a community-based postpartum clinic.

4. Determine the feasibility of operating this type of clinic in other settings across Ontario.

2.2 Hypothesis

We hypothesize that women discharged within 24 (±12) hours post vaginal delivery or 48 (±12) hours post-Caesarian section) and referred to the post-partum clinic will show a higher rate of exclusive breastfeeding at 3 months post-delivery than those who do not participate in this follow-up program (women from this study and from the previous pilot study). Our secondary hypothesis relates to the use of resources and the economic impact of the clinic. We believe that there will be fewer neonatal emergency room visits, fewer hospital readmissions and an overall decrease in the use of hospital and healthcare resources from the new mothers attending the breastfeeding clinic. This will lower the costs associated with these resources and help increase bed availability at hospitals and medical centers. We also hypothesize that women attending the clinic will be more satisfied and that the sustainability of this clinic and transferability to other settings will be feasible.

2.3 Primary Outcome

- The rate of exclusive breastfeeding at 3 months post-birth. We define exclusive breastfeeding as the feeding of the infant's mother's milk only for at least 2 weeks prior to collected outcome.

2.4 Secondary Outcomes

1. Rates of hyperbilirubinemia requiring phototherapy or hospitalization
2. Poor infant weight gain requiring intervention (pumping, supplementation, hospitalization)
3. Incidence of breastfeeding difficulties (e.g., sore nipples, insufficient milk supply, inadequate latch, engorgement)
4. Breastfeeding self efficacy score (Breastfeeding Self Efficacy Scale or BSES) at 2 weeks, 1 month and 3 months
5. Score of the Edinburgh Post-partum Depression Scale for new mothers
6. Access to community-based services: Public Health Clinics, Family Doctor, Pediatrician
7. Number of emergency department visits for the mother and the baby
8. Number of hospital readmissions for the mother and her baby
9. Mothers' satisfaction with the support received
10. Length of stay in the hospital, from delivery to discharge
11. Costs associated with this community-based postpartum clinic.

2.5 Inclusion criteria:

Mothers having delivered a baby at The Ottawa Hospital - General or Civic Campus:

1. Who are ≥ 18 years at the time of enrollment
2. Who are any parity, with a singleton infant born > 36 + 6 weeks of Gestational Age
3. Who have no medical counter indication for discharge at 24 (±12) hours post vaginal delivery or 48 (±12) hours post C-section delivery and for which the physician (family physician or obstetrician) has agreed that the mother is eligible
4. Who are breastfeeding and intend to breastfeed their baby upon discharge
5. Whose infant is healthy with no counter indication for discharge at 24 (±12) hours post vaginal birth or 48 (±12) hours post C-section birth and for which the physician (family physician or paediatrician) has agreed that the infant is eligible
6. Who can be contacted by phone or E-mail after hospital discharge

2.6 Exclusion criteria:

Mothers:

1. Who have had breast surgery
2. Who do not understand French or English
3. Who are unable to present to the clinic (transport not available)
4. Who have birthed multiples or preterm
5. Whose infants are exclusively formula-fed
6. Who are adoptive mothers
7. Who have been identified with a psychological risk that may impede her ability to attend the first appointment at the clinic

3 Methods:

3.1 Participant recruitment and informed consent

Upon arrival flyers (see section 8.9) will be handed out by a healthcare professional to eligible mothers. Mothers who have read the flyer and are interested in discussing the study will inform their healthcare provider or contact the research coordinator to speak with the study staff. If a woman declines to meet with the study staff, there will be no further contact.

Women who agree to meet with the research staff but are then found to be ineligible or decline to participate will not be enrolled in the study and will not be contacted further. However, the research staff will record the date of the meeting with the participant and the reason for not participating in the study.

A consent form will be given to the participant in either French or English. Participants will be asked for their contact information what time of day is best to contact them.

3.2 Events prior to hospital discharge

3.2.1 Data collection

Once consent has been obtained, the research team will collect information about delivery (mode of delivery and date and time of birth), obstetrical (previous pregnancies, parity) history, and information on the baby (gender, ethnicity, gestational age at birth, birth weight, Apgar score, bilirubin levels (TCB [trans cutaneous bilirubin] and/or TSB [total serum bilirubin), whether supplementation was given and date and time of discharge) from the hospital chart.

Contact information will be recorded in the study master log. As well, the OHIP number will be recorded in the master log to eventually allow the linkage of the dataset to ICES administrative data.

Upon confirmation by the pediatrician, the obstetrician, the family physician, and/or the midwife in attendance that the mother and baby have no counter-indication for early discharge, the research team will proceed to randomize the participant.

3.2.2 Randomization

Study participants will be randomly assigned to either the intervention (discharge with follow-up at the breastfeeding clinic) or the control (standard of care) groups via a 2:1 allocation ratio, with group designations being determined after the patient is enrolled and informed consent is obtained. Stratification will be used to improve allocation balance by site of recruitment and parity of participants. Group designation is given from a randomization list, which will be generated using a permuted randomized block design, with permuted blocks of sizes 3, 6, and 9 units, prior to study initiation. This list is then entered directly into REDCap's group allocation component which assigns group placement based on the inputted randomization list at the time of initial data entry, all the while blinding the study researchers, recruiters, and participants to the randomization allocations prior to patient enrollment and data collection. The study staff will then inform the participant of their randomization group. Rather than occurring at the time of consent, randomization will be delayed until confirmation by the nurses and/or attending physicians that the participant is indeed a candidate for discharge at approximately 24h (± 12) after vaginal or 48h (± 12) after C section delivery.

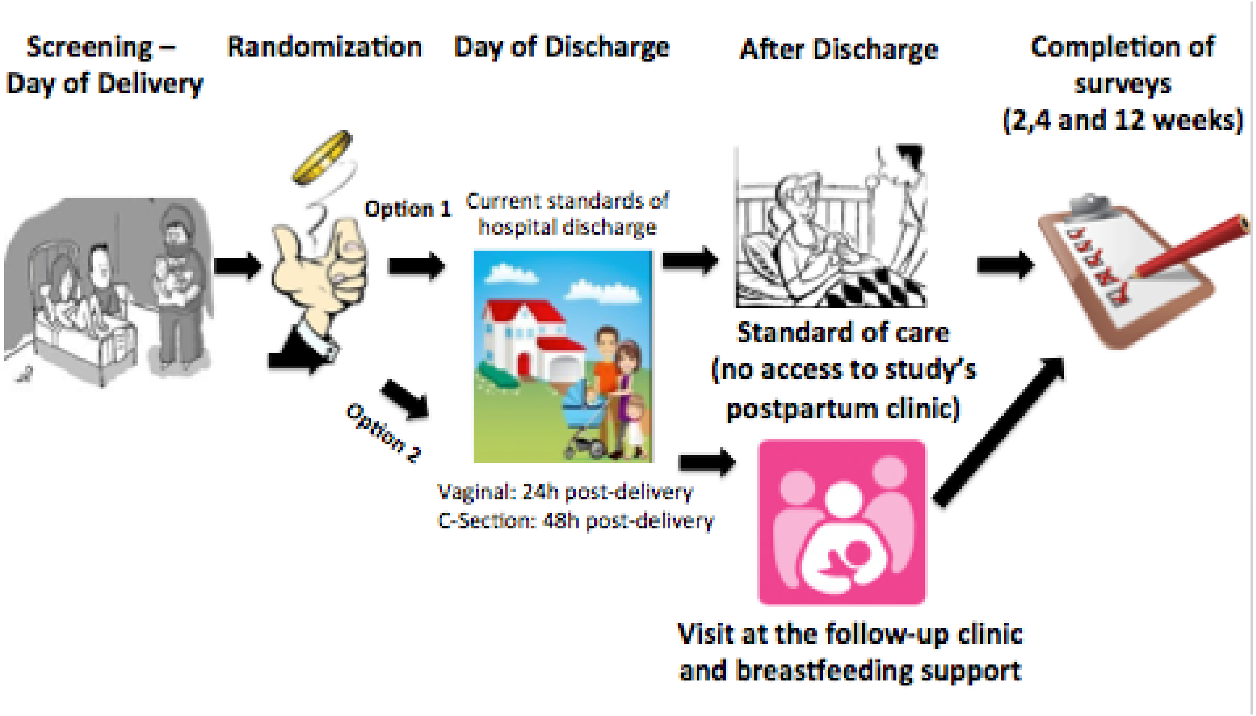

FIGURE 1. Schematic diagram showing the two possible outcomes after randomization. In the case of this breastfeeding study, the participants may be assigned the standard-care or the post-partum clinic option.

3.2.3 Appointment booking and additional information given to the participant (intervention arm)

Prior to patient discharge, an appointment will be made at the clinic. Participants will be provided with additional information on clinic location through a brochure (see section 8.8) prior to discharge. The brochure will also contain a reminder of the time of their appointment. The Monarch Clinic will be found at Harmony Medical Centre, 152 Cleopatra Drive, located south-west of downtown, a 15 minute drive from

the Civic campus and 20 minutes from the General campus. The Harmony Clinic has abundant parking (free on the street or a small fee inside the associated lot) and is fully accessible.

3.3 Non-intervention arm post-discharge events

Women randomly assigned to the non-intervention arm option will be discharged according to the current hospital standards, in accordance with their physician or midwife's decision. After hospital discharge, the participant and her baby will be entitled to receive follow-up care and seek breastfeeding support that is currently available in the community (e.g. through their family doctor, Public Health Unit or private services), but may not attend the pilot breastfeeding clinic.

Women will be contacted at 2 weeks, 1 month and 3 months post-delivery to complete the different surveys and questionnaires (3.5).

As is current standard of care practice, women will be assessed by Public Health Nurses for post-partum depression shortly after birth. Services will be provided as per the standard.

As part of the research, women will be contacted by phone at 3 weeks by a Public Health Nurse to administer the Edinburgh Postnatal Depression Scale. In the event of a high score, the Ottawa Public Health Nurse will provide the participant with appropriate support. Information collected will be abstracted by the research staff.

3.4 Intervention arm post-discharge events

Women randomly assigned to the intervention will be discharged approximately 24 hours following a vaginal delivery or approximately 48 hours following a C-section. While this is the aimed ideal time of discharge, there may be variation as time of discharge will ultimately depend on the attending physician/midwife's opinion/orders and what he/she feels is in the best interest of the mother and her newborn. Women who choose to stay in the hospital longer will still be referred to the Monarch Clinic and will not be excluded from the study.

Women and their infants will be expected to attend a pre-booked appointment at the breastfeeding clinic, scheduled within 48 hours of their discharge. Clinic staff will follow up with the participant if she fails to keep the mandatory follow-up appointment. This first appointment will include maternal assessment and care (e.g., wound care, prescriptions), neonatal care (e.g., tongue-tie clips, jaundice screening using transcutaneous bilirubinometer), blood work including TSB (samples couriered to TOH central laboratory, HBHC postpartum assessment, and breastfeeding assessment and support. Family physicians will be available for on-site consultations in the mornings, and lactation consultants and registered nurses will be at the clinic throughout the day from Monday to Friday and Saturday mornings. Medical data (e.g., bilirubin level (transcutaneous and serum), newborn assessment, infant weight gain, urination and defecation frequency, breastfeeding assessment and progress, maternal care) found in the clinic chart will be gathered.

The Bilirubin Pathway, established by the CMNRP, will be used to guide for the management of neonatal jaundice. Those guidelines provide recommendation for subsequent TSB and or TCB follow-up, according to the modified Buthani's normogram and whether risk factors for hyperbilirubinemia are

present or not. For babies discharged before 24 hours, blood for the newborn screening will be drawn at the first visit and sent by mail to the Newborn Screening Ontario laboratory located at CHEO.

Additional follow-up visits at the breastfeeding clinic will be made available to participants for the month following the birth of their baby.

Participants in the intervention group will also be contacted at 2 weeks, 1 month and 3 months to complete the different surveys and questionnaires (3.5).

Women will be assessed by a Public Health Nurse for post-partum depression in the clinic. Services will be provided as per the standard.

As part of the research, women will be contacted by phone at 3 weeks by a nurse from Ottawa Public Health to administer the Edinburgh Postnatal Depression Scale**.** In the event of a high score, the Ottawa Public Health Nurse will provide the participant with appropriate support. Information collected will be abstracted by the research staff.

3.5 Follow-up surveys at 2 weeks, 1 month, 3 months and 6 months after hospital discharge

Participants will be contacted by email or by phone to complete the following:

- At 2 weeks:
    - **A questionnaire assessing breastfeeding complications**, developed for this study
    - **Breastfeeding Self Efficacy Scale** (BSES-SF). This is a 14-item self-report instrument developed to measure breastfeeding confidence [14]
    - A questionnaire to assess the **access to community resources**, the rate of readmission of the babies and the level of satisfaction of mothers with the breastfeeding support received.
    - A questionnaire to determine the **rate of exclusive breastfeeding**. Participants will be asked to answer question on the methods used to feed their babies in the past 24 h hours and in the past two weeks. Exclusive breastfeeding will be as defined as the feeding of only the infant's mother's milk for at least 2 weeks prior to collected outcome.

- At 3 weeks only (by phone by a nurse from Ottawa Public Health)
    - **Edinburgh Postnatal Depression Scale.** This is a 10-item screening tool to identify postpartum depression at the time of its administration [13]. A score of 13 or higher on the EPDS is considered indicative of postpartum depression and a score of 10 to 12 is indicative of being at risk for postpartum depression (see section 3.6).

- At 1 month:
    - **Breastfeeding Self Efficacy Scale** (BSES-SF). This is a 14-item self-report instrument developed to measure breastfeeding confidence [14]

- A questionnaire to assess the **access to community resources**, the rate of readmission of the babies and the level of satisfaction of mothers with the breastfeeding support received.
- A questionnaire to determine the **rate of exclusive breastfeeding**. Participants will be asked to answer question on the methods used to feed their babies in the past 24 h pours and in the past two weeks. Exclusive breastfeeding will be as defined as the feeding of only the infant's mother's milk for at least 2 weeks prior to collected outcome.
- **Socio-demographics survey**. This survey was developed using questions and categories used in the Public Health Agency of Canada survey "What Mothers Say: The Canadian Maternity Experiences " [12]

- At 3 months:
  - **Breastfeeding Self Efficacy Scale** (BSES-SF). This is a 14-item self-report instrument developed to measure breastfeeding confidence [14]
  - A questionnaire to assess the **access to community resources**, the rate of readmission of the babies and the level of satisfaction of mothers with the breastfeeding support received.
  - A questionnaire determine the **rate of exclusive breastfeeding**. Participants will be asked to answer question on the methods used to feed their babies in the past 24 h pours and in the past two weeks. Exclusive breastfeeding will be as defined as the feeding of only the infant's mother's milk for at least 2 weeks prior to collected outcome.
  - **Mother Satisfaction Survey** (developed for this study).

- At 6 months:
  - Follow-up survey: A very short survey (5 minutes) to determine breastfeeding habits at 6 months.

All surveys (in French and English) are included in section 8 (the surveys are the same as the ones used in the pilot study, except for the addition of the Mother Satisfaction Survey). Participants will be offered the possibility to complete the follow-up surveys online. The master study log will include the name of the participant, their full contact information including email address, and their study ID number. The links to the online questionnaires, instructions and login information will be sent by email (to participants who provide their email address) at 15 days, 1 month, 3 months and 6 months. If the participants do not complete the questionnaires within 2 days, we will send them a reminder by email. Offering to complete the questionnaires online will give flexibility to the participants providing them with the opportunity to complete the survey at their own pace. Participants will have the ability to save their answers without completing the totality of the survey and return to the questionnaire later, for extra flexibility given the unpredictable nature of a mother's schedule.

Participants will also be offered the option to be contacted over the phone by the research staff to complete the survey, as some may not have internet access or be comfortable answering an electronic

survey. The entire phone interview should take approximately 40 minutes at each contact. Recognizing that the first few weeks after returning home with a newborn can be overwhelming for the mother, the interview may be done in multiple shorter sessions, at the mother's discretion, to accommodate her.

In the event of a high score on the Edinburgh Postnatal Depression Scale (indicating possible mood disorder), the Ottawa Public Health Nurse will provide the participant with appropriate support and referrals.

The anticipated duration of the study is 11 months, and ends with the completion of the last set of surveys (5 months of recruitment + 6 months of follow-up).

3.6 Risks and risk management

There is no anticipated risk to the participant or her baby for participating in this trial. In the event that participants or their babies become ill or experience difficulties prior to their scheduled appointment at the clinic, they will be reminded to go to the emergency department of consult with their family doctor, and not wait for the appointment if they feel the problem is urgent. Given that hospital discharge 24 hours after vaginal delivery and 48 hours after C-section is not an uncommon practice in hospital settings, we feel that the risks for this study are low.

In the event that a participant randomized to the intervention group needs to stay in hospital longer than anticipated, due to their attending physician's decision, she will still have access to the postpartum clinic post discharge. Participants will be analyzed as "intent to treat" events.

As stated in the Information and Consent Form, participants can decline answering any question.

3.7 Criteria for removal/withdrawal from the study

Participants may decide to withdraw from the study at any time point in the study. A participant may be removed from the study if the baby or the mother becomes gravely ill and needs intensive care or if the attending physician feels that it is in the best interest for the mother and baby not to continue with the study. If withdrawn for the study, the researcher will still use the data already collected from the participant unless she asks for it to be destroyed. No new information will be collected.

3.8 Study calendar

**Timing** / **Events**	Day of Delivery - Screening (Day 0)	Day after discharge (Day 1-2)	Between Day 1-Day 30 (Additional Visits-Optional)	Day 14	Day 21	Day 30	Day 84	Day 180
Informed consent	X S							
Eligibility Confirmation	X S							
Ask for contact information	X S							
Randomization	X S							
Collection of information about delivery, obstetrical and medical history and	X S							

information on the baby from hospital chart								
Hospital discharge within 24 hours for vaginal birth and 48 hours for C-section	X							
Maternal care and neonatal care (jaundice screening using transcutaneous bilirubinometer and weight gain assessment) at the clinic.		X						
Breastfeeding support at clinic		X	X					
Additional medical care at clinic			X					
Collection of medical data from visits at clinic		X	X					
Demographics survey						X S		
Edinburgh Postnatal Depression Scale					X S	X S		
Breastfeeding local complications survey				X S		X S		
Breastfeeding survey				X S		X S	X S	
Use of resources survey				X S		X S	X S	
Self-efficacy survey				X S		X S	X S	
Mother Satisfaction Survey							X S	
6 month follow up survey								X S

Index: X = Women assigned to the breastfeeding clinic program will be completing these events
S = Women assigned to the standard care will be completing these events

4 Statistics

4.1 Sample size

Based on an estimated 50% rate of exclusive breastfeeding at 3 months in the control group, 200 patients per group will be needed to detect a 15% relative difference in the intervention group (α=0.05, β=0.9, one-sided test). Assuming an attrition rate of 15% (i.e. loss to follow up, unsuitability, or other unanticipated events), we set a target of **230 participants per group** making a total of **460** women. Accounting for the logistical concern of making full use of the nursing staff, lactation consultants, and family physicians at the clinic, we decided to apply a 1:2 ratio for control to intervention allocations and determined that 154 and 306 patients are needed for the control and intervention groups respectively (maintaining significance level, power, and effect size).

4.2 Feasibility

It is estimated that the number of deliveries at our catchment hospitals is approximately 3200/year at each site. Assuming that at least 55% of those women qualify for the study, there will be an average of 10 eligible patients/day during the study period. We anticipate a response rate of 50% and expect to

enroll an average of 5 women per day, 6 days a week. The anticipated total number of patients who qualify to be enrolled in the study is 5 patients x 6 days x 4 weeks x 5 months = 600 (Figure 2). Therefore, even with unexpected events or lower than expected daily recruitment capacity our final estimated sample sizes should be sufficient to recruit enough participants for the on-time completion (8 months) of the study.

Figure 2: Flow chart of expected study participant recruitment

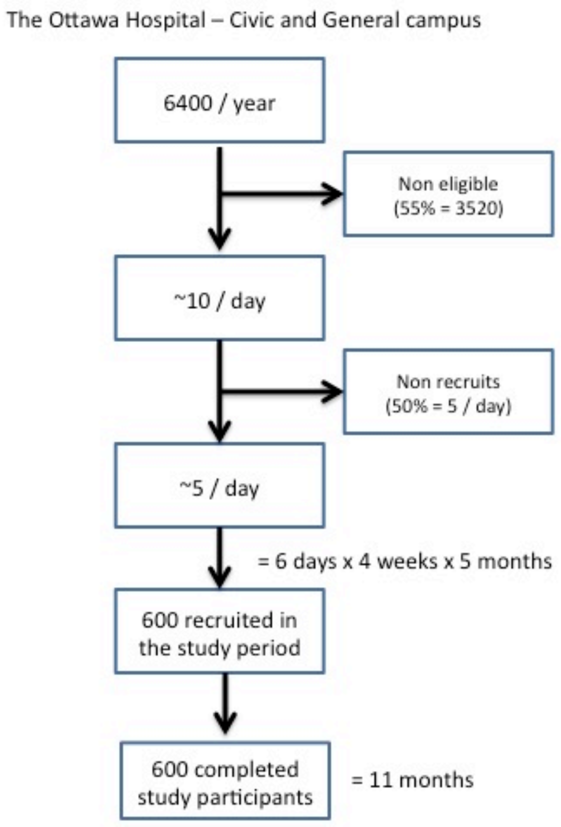

4.3 Statistical analysis

All outcomes will be analyzed based on the intention-to-treat principle, in accordance with the randomization allocations described previously. The primary goal of this study is to test the hypothesis that rates of exclusive breastfeeding at 3 months (defined as the feeding of only the infant's mother's milk for at least 2 weeks prior to collected outcome) will be higher in the group of women attending the post-partum clinic, as compared to those under standard of care. For comparison between intervention and control arms we will employ a generalized estimating equation multivariable regression model to account for prognostic risk factors that have not been sufficiently balanced by randomization. Group and individual level covariates will be included in the model, with recruitment site as the clustering unit to allow for inference at the individual level. This design will also investigate the effect of demographic

drivers such as maternal age, education, parity, etc., on the primary outcome, producing both crude and adjusted risk estimates and their 95% confidence intervals.

Secondary outcomes will be collected by studying the medical data (such as rates of hyperbilirubinemia and self-efficacy) that will be entered in the database by the research staff. This data will be compared across case and control groups using the Pearson Chi-squared, Student's t, or Fisher's exact test, depending on their observed frequencies. We will also compare the statistics and outcomes from the present study with baseline estimates from phase 1. The answers women provide through their questionnaires and surveys will be the main instruments for determining exclusive breastfeeding rates and their behavioral and emotional measurements. For the cost-effectiveness analysis, direct and indirect costs for the intervention and control groups will be compared regarding use of resources (e.g., clinic visits, physician visits, Emergency Department visits, hospital readmission, and indirect cost).

5 Data management

5.1 Data collection

Data collected from participant charts and during phone interview will be recorded by research personnel in a web-based Case Report Form created using the secure platform: REDCap. Each participant will be identified by a unique ID number.

Details regarding delivery, obstetrical history, and information on the baby will be abstracted from the charts.

Information from the clinic visits including referrals will be abstracted from the clinic charts.

Data will also be collected by the means of surveys sent to the mother. Participants completing surveys online will receive a link by E-mail to complete the surveys. Upon clicking on the "submit" button the survey will be sent to research database and will no longer be available to the participant.

5.2 Database security

REDCap (Research Electronic Data Capture) is a secure, web-based application designed exclusively to support data capture for research studies. REDCap provides: 1) an intuitive interface for data entry (with data validation); 2) 128 bit encryption between the data entry client and the server (https); 3) audit trails for tracking data manipulation and export procedures; 4) automated export procedures for seamless data downloads to common statistical packages (SPSS, SAS, Stata, R); 5) procedures for importing data from external sources; and 6) advanced features, such as branching logic and calculated fields.

REDCap is developed and maintained by a team at Vanderbilt University and licensed free of charge by the Research Institute at the Children's Hospital of Eastern Ontario (CHEO). The application and data are housed on servers provided by CHEO. These servers are located within CHEO's secure data center. Local support for REDcap is provided by CHEO's Clinical Research Unit.

CHEO's Clinical Research Unit Data Coordinating Centre (DCC) will be used as a central location for data processing and management. The DCC will house the data in a dedicated, locked server room within the CHEO Hospital main site, which is secured with limited badge access security cameras and 24-hour on-site security guards. The DCC coordinates its network infrastructure and security with CHEO Information Systems (CHEO IS). This provides the DCC with segregated and redundant firewalls and switches, HVAC, malware and anti-virus support, data backup and recovery support, and server hardware and software support within CHEO IS. Network equipment includes two servers connected to redundant gigabit switches. Administrative user authentication to the servers is centralized to CHEO IS Active Directory while the application authentication uses an internal table-based authentication method. Communication over public networks and between the web application and the database is encrypted using secure socket layer (SSL) with 256-bit encryption or higher.

For participants who prefer electronic follow-up, a link to a secure, web-based questionnaire will be sent to a personal email address provided by the participant. E-mail addresses will be entered and stored directly in the study database on a discrete instrument (page) within the project. Access to this page will be restricted to study team members who are responsible for inputting the address and sending the questionnaire. Any study team member who requires access to the dataset but does not need to view/input the e-mail address will be assigned "No Access" to the page. E-mail addresses will not be printed or exported at any time. Furthermore, all study team members will be granted "De-Identified" access for Data Export to ensure that e-mail addresses are never exported. As with all data entered into the REDCap database, e-mail addresses will be stored on servers in CHEO's secure Data Center within the hospital main site.

The investigators and DCC staff are fully committed to the security and confidentiality of all research data. All DCC personnel have signed confidentiality agreements concerning all data encountered in the center. Violation of these agreements may result in termination from employment at the CHEO Research Institute. In addition, all personnel involved with DCC data systems have completed GCP training and are governed by DCC standard operating procedures that address data management, privacy and security

6 Ethical considerations

6.1 Ethics committee

The study may only be initiated after the Investigator has obtained written approval of the protocol, Informed Consent form and other study documents, and any amendments (if applicable), by the Ottawa Health Science Network Research Ethics Board (OHSN-REB). Changes in protocol (amendments) must be submitted to the OHSN-REB for approval. The Investigator will submit reports on, and reviews of, the trial and its progress to the OHSN-REB at the intervals according to their guidelines. The principal investigators (TLM and MW) will review each of the cases requiring hospital readmission within 15 days after discharge, and after each 200 new enrolled patients, a summary of the recruitment and the number of readmission will be forwarded to a Data Safety Monitoring Board (DSMB). The DSMB will consist of one statistician, one paediatrician (or neonatologist), and one family physician with expertise in obstetrics.

6.2 Participant information and consent

The Investigator or study staff will explain to each potential participant the aims, methods, reasonably anticipated benefits and potential risks of this study in written form and by verbal explanation in non-technical terms (lay language). After this explanation and prior to any trial related activity, participants will give their informed consent by completing the consent form. Both the participant and the Investigator (or study staff) must sign and date the form. The participant will receive a copy of the completed informed consent. The participants assigned to the clinic will receive a brochure with information on the location of the clinic and details of their appointment time.

6.3 Compensation

Women randomized to the intervention arm of the study will be reimbursed $20 plus one parking token per visit (to a maximum of three visits) for costs associated with travel to the clinic.

6.4 Records retention

The trial related records will be retained for 10 years. Records will be kept to enable linkage of participants' identify to CRF data (master log). This includes sufficient information from hospital and clinic records such as original signed informed consent forms, contact information and completed surveys. After 10 years, all study records will be destroyed.

7 Reference List

(1) City of Ottawa PH. Infant Care Survey. 2005. Ottawa, City of Ottawa, Public Health Unit.

(2) Public Health Agency of Canada. What Mothers Say: The Canadian Maternity Experiences Survey. 173-177. 2009. Ottawa, PHAC.

(3) Paricio Talayero J, Lizan-Garcia M, Otero Puime A, et al. Full breastfeeding and hospitalization as a result of infections in the first year of life. Pediatrics 2006; 118:e92-e99.

(4) Bachrach VR, Schwarz E, Bachrach LR. Breastfeeding and the risk of hospitalization for respiratory disease in infancy: A meta-analysis. Arch Pediatr Adolesc Med 2003; 157:237-243.

(5) Harder T, Bergmann R, Kallischnigg G, et al. Duration of breastfeeding and risk of overweight: A meta-analysis. Journal of Epidemiology 2005; 162:397-403.

(6) Newcomb PA, Storer BE, Longnecker MP, et al. Lactation and a reduced risk of premenopausal breast cancer. New England Journal of Medicine 1994; 330:81-87.

(7) Collaborative Group on Hormonal Factors in Breast Cancer. Breast cancer and breastfeeding: Collaborative reanalysis of individual data from 47 epidemiological studies in 30 countries, including 50302 women with breast cancer and 96973 women without the disease. Lancet 2002; 360:187-195.

(8) Rosenblatt KA, Thomas DB. WHO Collaborative Study of Neoplasia and Steroid Contraceptives. International Journal of Epidemiology 1993; 22:192-197.

(9) Hatton, Daniel C., Jane Harrison-Hohner, Sarah Coste, Veronica Dorato, Luis B. Curet, and David A. McCarron. "Symptoms of postpartum depression and breastfeeding." *Journal of Human Lactation* 21, no. 4 (2005): 444-449.

(10) Dennis C. The Breastfeeding Self-Efficacy Scale: psychometric assessment of the short form. JOGNN: Journal of Obstetric, Gynecologic, and Neonatal Nursing 32(6): 734-44, 2003 Nov-Dec (49 ref) 2003;(6): 734-744.

(11) Renfrew MJ, McCormick FM, Wade A, Quinn B, Dowswell T. Support for healthy breastfeeding mothers with healthy term babies. Cochrane Database of Systematic Reviews 2012; 2012(5. Art.No: CD001141.DOI: 10.1002/14651858.CD001141.pub4.).

(12) Public Health Agency of Canada. What Mothers Say: The Canadian Maternity Experiences Survey. Ottawa, 2009.

(13) Cox JL, Holden JM, Sagovsky R. Detection of postnatal depression. Development of the 10-item Edinburgh Postnatal Depression Scale. Br J Psychiatry. 1987;150:782–6.

(14) Denis CL The Breastfeeding Self-Efficacy Scale: Psychometric Assessment of the Short Form J OGNN, 32, 734–744; 2003. DOI: 10.1177/0884217503258459

8 Evaluation tools

8.1 Demographics

8.2 Edinburgh Post Partum Depression Scale

8.3 Breastfeeding Self Efficacy Scale

8.4 Local Breastfeeding Complications Questionnaire

8.5 Use of Resources Questionnaire

8.6 Breastfeeding Questionnaire

8.7 Mother Satisfaction Survey

8.8 Informative pamphlet

8.9 Recruitment flyer
